# *trans*-Di-μ-carbonyl-bis­{carbon­yl[η^5^-2,3,4,5-tetra­methyl-1-(2-thien­yl)cyclo­penta­dien­yl]ruthenium(I)}(*Ru*—*Ru*)

**DOI:** 10.1107/S1600536809026063

**Published:** 2009-07-11

**Authors:** Zhi-Hong Ma, Gui-Ying Dong, Xiao-Huan Liu, Jin Lin

**Affiliations:** a College of Chemistry and Materials Science, Hebei Normal University, Shijiazhuang 050016, People’s Republic of China; b College of Basic Medicine, Hebei Medical University, Shijiazhuang 050017, People’s Republic of China; c College of Chemical Engineering and Biotechnology, Hebei Polytechnic University, Tangshan 063009, People’s Republic of China

## Abstract

The title compound, [Ru_2_(C_13_H_15_S)_2_(CO)_4_], is a centrosymmetric binuclear metal–carbonyl complex containing an Ru—Ru single bond [2.7511 (8) Å]. Each Ru^I^ atom is coordinated by two bridging carbonyl ligands, one terminal carbonyl ligand and one η^5^-cyclo­penta­dienyl group. The complex has a *trans* conformation and the two cyclo­penta­dienyl ring planes are parallel. The crystal structure involves weak C—H⋯O hydrogen bonds.

## Related literature

For general background to substituted cyclo­penta­dien­yl–metal complexes, see: Arndt (2002 ▶); Bailey *et al.* (1978 ▶); King (1976 ▶); Möhring & Coville (2006 ▶). For the crystal structures of related ruthenium complexes, see: Schumann *et al.* (2002 ▶).
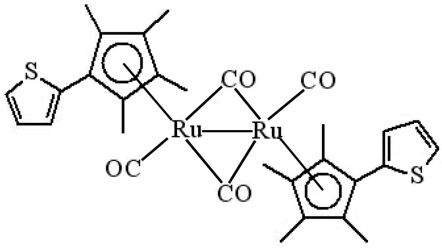


## Experimental

### 

#### Crystal data

[Ru_2_(C_13_H_15_S)_2_(CO)_4_]
*M* *_r_* = 720.82
Triclinic, 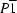

*a* = 8.269 (2) Å
*b* = 8.899 (3) Å
*c* = 10.056 (3) Å
α = 81.826 (4)°
β = 76.083 (5)°
γ = 82.876 (5)°
*V* = 707.9 (4) Å^3^
*Z* = 1
Mo *K*α radiation
μ = 1.25 mm^−1^
*T* = 273 K
0.15 × 0.12 × 0.10 mm

#### Data collection

Bruker SMART APEX CCD diffractometer
Absorption correction: multi-scan (*SADABS*; Sheldrick, 1996 ▶) *T*_min_ = 0.835, *T*_max_ = 0.885
3667 measured reflections
2493 independent reflections
2431 reflections with *I* > 2σ(*I*)
*R*_int_ = 0.016

#### Refinement

*R*[*F* ^2^ > 2σ(*F* ^2^)] = 0.027
*wR*(*F* ^2^) = 0.100
*S* = 1.03
2493 reflections
173 parameters
H-atom parameters constrained
Δρ_max_ = 0.53 e Å^−3^
Δρ_min_ = −0.63 e Å^−3^

### 

Data collection: *SMART* (Bruker, 2007 ▶); cell refinement: *SAINT* (Bruker, 2007 ▶); data reduction: *SAINT*; program(s) used to solve structure: *SHELXS97* (Sheldrick, 2008 ▶); program(s) used to refine structure: *SHELXL97* (Sheldrick, 2008 ▶); molecular graphics: *SHELXTL* (Sheldrick, 2008 ▶); software used to prepare material for publication: *SHELXTL*.

## Supplementary Material

Crystal structure: contains datablocks I, global. DOI: 10.1107/S1600536809026063/hy2205sup1.cif

Structure factors: contains datablocks I. DOI: 10.1107/S1600536809026063/hy2205Isup2.hkl

Additional supplementary materials:  crystallographic information; 3D view; checkCIF report

## Figures and Tables

**Table 1:** Selected bond lengths (Å)

Ru1—C1	2.018 (3)
Ru1—C1^i^	2.048 (3)
Ru1—C2	1.862 (3)
Ru1—C3	2.246 (3)
Ru1—C4	2.291 (3)
Ru1—C5	2.302 (3)
Ru1—C6	2.282 (3)
Ru1—C7	2.217 (3)

Symmetry code: (i) 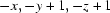.

**Table 2:** Hydrogen-bond geometry (Å, °)

*D*—H⋯*A*	*D*—H	H⋯*A*	*D*⋯*A*	*D*—H⋯*A*
C10—H10⋯O2^ii^	0.93	2.60	3.335 (5)	136
C14—H14*B*⋯O2^i^	0.96	2.58	3.319 (4)	134

Symmetry codes: (i) 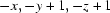; (ii) 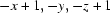.

**Fig. 1.**
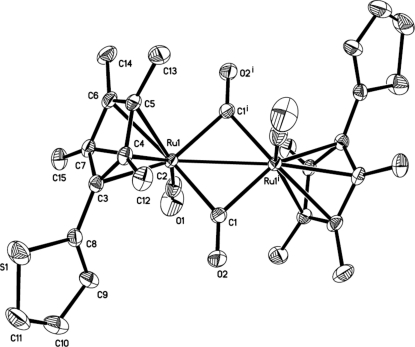
The molecular structure of the title compound. Displacement ellipsoids are drawn at the 30% probability level. H atoms have been omitted for clarity. [Symmetry code: (i) -x, 1-y, 1-z.]

## supplementary crystallographic information

### Comment

Cyclopentadienyl metal complexes have been extensively investigated since ferrocene has been discovered. Replacement of the hydrogen atoms by other substituents alters both the steric and electronic influences of the *H*^5^-cyclopentadienyl ring, resulting in differing reactivity and stability of the substituted cyclopentadienyl metal complexes (Arndt, 2002; King, 1976). Especially for metallocene polymerization catalysts, the steric and electronic effects of the substituents on cyclopentadienyl ring greatly influence catalytic activity (Bailey *et al.*, 1978; Möhring & Coville, 2006).

The title compound, [Ru_2_(C_13_H_15_S)_2_(CO)_4_], is a centrosymmetric binuclear metal–carbonyl complex containing an Ru—Ru single bond. As shown in Fig. 1, the cyclopentadienyl ring of the organic ligand coordinates to the Ru^I^ atom (Table 1), while the thienyl group acting as a substituent is uncoordinated. The Ru1—Cg1 distance is 1.911 (3) Å, where Cg1 is the centroid of the cyclopentadienyl ring. The Ru—Ru bond distance is 2.7511 (8) Å and agrees with that observed in the analogous structure [2.751 (1) Å] (Schumann *et al.*, 2002). The two cyclopentadienyl rings are parallel by virtue of the center of symmetry. The complex has a *trans* conformation, with two bridging carbonyl ligands and two terminal carbonyl ligands. The crystal packing is stabilized by weak C—H···O hydrogen bonds (Table 2).

### Experimental

A solution of 1-(2-thienyl)-2,3,4,5-tetramethylcyclopentadiene (0.288 g, 1.41 mmol) and Ru_3_(CO)_12_ (0.30 g, 0.47 mmol) in xylene (30 ml) was refluxed for 12 h. The solvent was removed under vacuum and the residue was chromatographed on an Al_2_O_3_ column using petroleum ether/dichloromethane (volume ratio = 1:3) as eluent. The red band was collected, and after several days red crystals were obtained (yield 0.142 g, 27.9%). Analysis calculated for C_30_H_30_O_4_Ru_2_S_2_: C 49.99, H 4.19%; found: C 49.94, H 4.21%.

### Refinement

H atoms were positioned geometrically and refined as riding atoms, with C—H = 0.93 (CH) and 0.96 (CH_3_) Å and with *U*_iso_(H) = 1.2(1.5 for methyl)*U*_eq_(C).

### Crystal data

**Table:**

[Ru_2_(C_13_H_15_S)_2_(CO)_4_]	*Z* = 1
*M**_r_* = 720.82	*F*(000) = 362
Triclinic, *P*1	*D*_x_ = 1.691 Mg m^−^^3^
Hall symbol: -P 1	Mo *K*α radiation, λ = 0.71073 Å
*a* = 8.269 (2) Å	Cell parameters from 1002 reflections
*b* = 8.899 (3) Å	θ = 4.5–22.2°
*c* = 10.056 (3) Å	µ = 1.25 mm^−^^1^
α = 81.826 (4)°	*T* = 273 K
β = 76.083 (5)°	Block, red
γ = 82.876 (5)°	0.15 × 0.12 × 0.10 mm
*V* = 707.9 (4) Å^3^	

### Data collection

**Table:**

Bruker SMART APEX CCD diffractometer	2493 independent reflections
Radiation source: fine-focus sealed tube	2431 reflections with *I* > 2σ(*I*)
graphite	*R*_int_ = 0.016
φ and ω scans	θ_max_ = 25.1°, θ_min_ = 2.3°
Absorption correction: multi-scan (*SADABS*; Sheldrick, 1996)	*h* = −9→9
*T*_min_ = 0.835, *T*_max_ = 0.885	*k* = −10→10
3667 measured reflections	*l* = −11→9

### Refinement

**Table:**

Refinement on *F*^2^	Secondary atom site location: difference Fourier map
Least-squares matrix: full	Hydrogen site location: inferred from neighbouring sites
*R*[*F*^2^ > 2σ(*F*^2^)] = 0.027	H-atom parameters constrained
*wR*(*F*^2^) = 0.100	*w* = 1/[σ^2^(*F*_o_^2^) + (0.09*P*)^2^ + 0.0001*P*] where *P* = (*F*_o_^2^ + 2*F*_c_^2^)/3
*S* = 1.03	(Δ/σ)_max_ < 0.001
2493 reflections	Δρ_max_ = 0.53 e Å^−^^3^
173 parameters	Δρ_min_ = −0.63 e Å^−^^3^
0 restraints	Extinction correction: *SHELXL97* (Sheldrick, 2008), Fc^*^=kFc[1+0.001xFc^2^λ^3^/sin(2θ)]^-1/4^
Primary atom site location: structure-invariant direct methods	Extinction coefficient: 0.026 (3)

### Fractional atomic coordinates and isotropic or equivalent isotropic displacement parameters (Å^2^)

**Table:**

	*x*	*y*	*z*	*U*_iso_*/*U*_eq_	
Ru1	−0.01450 (2)	0.40733 (2)	0.623212 (18)	0.02438 (17)	
S1	0.40230 (13)	0.12210 (13)	0.86000 (11)	0.0622 (3)	
O1	−0.1870 (5)	0.1684 (4)	0.5431 (3)	0.0836 (10)	
O2	0.2945 (3)	0.3491 (3)	0.4011 (3)	0.0510 (6)	
C1	0.1653 (3)	0.4142 (3)	0.4472 (3)	0.0314 (6)	
C2	−0.1213 (4)	0.2620 (4)	0.5688 (3)	0.0440 (8)	
C3	0.1390 (3)	0.2962 (3)	0.7731 (3)	0.0298 (6)	
C4	0.1719 (3)	0.4517 (3)	0.7456 (3)	0.0302 (6)	
C5	0.0180 (4)	0.5430 (3)	0.7921 (3)	0.0327 (6)	
C6	−0.1104 (4)	0.4436 (3)	0.8499 (3)	0.0337 (6)	
C7	−0.0383 (4)	0.2915 (4)	0.8363 (3)	0.0319 (6)	
C8	0.2643 (4)	0.1630 (3)	0.7537 (3)	0.0329 (6)	
C9	0.2855 (4)	0.0553 (4)	0.6641 (4)	0.0452 (8)	
H9	0.2217	0.0550	0.5996	0.054*	
C10	0.4219 (5)	−0.0577 (4)	0.6847 (5)	0.0606 (10)	
H10	0.4556	−0.1393	0.6328	0.073*	
C11	0.4936 (5)	−0.0364 (5)	0.7814 (5)	0.0657 (11)	
H11	0.5834	−0.0992	0.8050	0.079*	
C12	0.3402 (4)	0.5088 (4)	0.6853 (3)	0.0423 (7)	
H12A	0.3276	0.6035	0.6280	0.063*	
H12B	0.4104	0.4352	0.6308	0.063*	
H12C	0.3905	0.5244	0.7583	0.063*	
C13	−0.0043 (5)	0.7128 (4)	0.7947 (4)	0.0500 (8)	
H13A	−0.0294	0.7352	0.8885	0.075*	
H13B	−0.0946	0.7558	0.7523	0.075*	
H13C	0.0970	0.7558	0.7449	0.075*	
C14	−0.2858 (4)	0.4923 (5)	0.9167 (3)	0.0504 (9)	
H14A	−0.3539	0.4098	0.9259	0.076*	
H14B	−0.3272	0.5783	0.8610	0.076*	
H14C	−0.2900	0.5202	1.0063	0.076*	
C15	−0.1239 (4)	0.1515 (4)	0.8928 (3)	0.0439 (8)	
H15A	−0.1240	0.1282	0.9890	0.066*	
H15B	−0.0661	0.0681	0.8443	0.066*	
H15C	−0.2372	0.1677	0.8818	0.066*	

### Atomic displacement parameters (Å^2^)

**Table:**

	*U*^11^	*U*^22^	*U*^33^	*U*^12^	*U*^13^	*U*^23^
Ru1	0.0253 (2)	0.0278 (2)	0.0202 (2)	−0.00256 (12)	−0.00515 (12)	−0.00352 (12)
S1	0.0550 (6)	0.0681 (7)	0.0699 (7)	0.0124 (5)	−0.0345 (5)	−0.0105 (5)
O1	0.121 (3)	0.074 (2)	0.071 (2)	−0.060 (2)	−0.0269 (19)	−0.0075 (16)
O2	0.0391 (13)	0.0660 (17)	0.0375 (13)	0.0190 (12)	−0.0024 (10)	−0.0029 (11)
C1	0.0284 (14)	0.0381 (15)	0.0274 (14)	0.0025 (11)	−0.0075 (11)	−0.0057 (12)
C2	0.056 (2)	0.0469 (18)	0.0334 (17)	−0.0202 (16)	−0.0128 (14)	−0.0008 (14)
C3	0.0289 (14)	0.0367 (14)	0.0258 (14)	−0.0035 (11)	−0.0108 (11)	−0.0023 (11)
C4	0.0323 (14)	0.0360 (15)	0.0242 (13)	−0.0065 (11)	−0.0093 (11)	−0.0020 (11)
C5	0.0397 (15)	0.0379 (16)	0.0235 (14)	0.0006 (12)	−0.0123 (11)	−0.0088 (12)
C6	0.0344 (15)	0.0460 (17)	0.0212 (13)	−0.0002 (13)	−0.0070 (11)	−0.0070 (12)
C7	0.0310 (14)	0.0416 (17)	0.0211 (14)	−0.0071 (12)	−0.0033 (11)	0.0017 (12)
C8	0.0322 (14)	0.0367 (15)	0.0290 (14)	−0.0025 (11)	−0.0091 (11)	0.0013 (12)
C9	0.0489 (19)	0.0351 (17)	0.0497 (19)	0.0096 (14)	−0.0128 (15)	−0.0076 (14)
C10	0.056 (2)	0.045 (2)	0.077 (3)	0.0101 (17)	−0.009 (2)	−0.0149 (19)
C11	0.046 (2)	0.058 (2)	0.086 (3)	0.0160 (18)	−0.019 (2)	0.005 (2)
C12	0.0353 (16)	0.0540 (19)	0.0393 (17)	−0.0175 (14)	−0.0081 (13)	−0.0001 (14)
C13	0.068 (2)	0.0416 (18)	0.0459 (19)	0.0010 (15)	−0.0186 (16)	−0.0176 (15)
C14	0.0393 (18)	0.073 (2)	0.0341 (17)	0.0036 (16)	0.0012 (14)	−0.0136 (17)
C15	0.0439 (18)	0.0474 (19)	0.0400 (17)	−0.0165 (15)	−0.0109 (14)	0.0095 (14)

### Geometric parameters (Å, °)

**Table:**

Ru1—C1	2.018 (3)	C6—C7	1.421 (4)
Ru1—C1^i^	2.048 (3)	C6—C14	1.484 (4)
Ru1—C2	1.862 (3)	C7—C15	1.483 (4)
Ru1—C3	2.246 (3)	C8—C9	1.374 (4)
Ru1—C4	2.291 (3)	C9—C10	1.449 (5)
Ru1—C5	2.302 (3)	C9—H9	0.9300
Ru1—C6	2.282 (3)	C10—C11	1.300 (6)
Ru1—C7	2.217 (3)	C10—H10	0.9300
Ru1—Ru1^i^	2.7511 (8)	C11—H11	0.9300
S1—C11	1.718 (4)	C12—H12A	0.9600
S1—C8	1.718 (3)	C12—H12B	0.9600
O1—C2	1.139 (4)	C12—H12C	0.9600
O2—C1	1.173 (3)	C13—H13A	0.9600
C1—Ru1^i^	2.048 (3)	C13—H13B	0.9600
C3—C4	1.420 (4)	C13—H13C	0.9600
C3—C7	1.454 (4)	C14—H14A	0.9600
C3—C8	1.476 (4)	C14—H14B	0.9600
C4—C5	1.436 (4)	C14—H14C	0.9600
C4—C12	1.499 (4)	C15—H15A	0.9600
C5—C6	1.428 (4)	C15—H15B	0.9600
C5—C13	1.502 (4)	C15—H15C	0.9600
			
C2—Ru1—C1	92.57 (13)	C6—C5—C13	124.5 (3)
C2—Ru1—C1^i^	93.27 (14)	C4—C5—C13	126.8 (3)
C1—Ru1—C1^i^	94.85 (10)	C6—C5—Ru1	71.09 (15)
C2—Ru1—C7	93.67 (13)	C4—C5—Ru1	71.36 (15)
C1—Ru1—C7	135.88 (11)	C13—C5—Ru1	128.18 (19)
C1^i^—Ru1—C7	128.26 (11)	C7—C6—C5	107.8 (2)
C2—Ru1—C3	110.26 (13)	C7—C6—C14	126.8 (3)
C1—Ru1—C3	99.57 (11)	C5—C6—C14	125.4 (3)
C1^i^—Ru1—C3	151.61 (11)	C7—C6—Ru1	69.11 (15)
C7—Ru1—C3	38.01 (10)	C5—C6—Ru1	72.61 (15)
C2—Ru1—C6	113.52 (13)	C14—C6—Ru1	125.5 (2)
C1—Ru1—C6	151.57 (11)	C6—C7—C3	108.2 (3)
C1^i^—Ru1—C6	94.73 (11)	C6—C7—C15	125.8 (3)
C7—Ru1—C6	36.80 (10)	C3—C7—C15	125.7 (3)
C3—Ru1—C6	61.88 (10)	C6—C7—Ru1	74.10 (16)
C2—Ru1—C4	146.49 (13)	C3—C7—Ru1	72.09 (15)
C1—Ru1—C4	91.03 (11)	C15—C7—Ru1	125.5 (2)
C1^i^—Ru1—C4	119.61 (11)	C9—C8—C3	129.5 (3)
C7—Ru1—C4	61.83 (10)	C9—C8—S1	111.3 (2)
C3—Ru1—C4	36.45 (10)	C3—C8—S1	119.1 (2)
C6—Ru1—C4	61.06 (10)	C8—C9—C10	110.1 (3)
C2—Ru1—C5	149.83 (12)	C8—C9—H9	125.0
C1—Ru1—C5	116.93 (11)	C10—C9—H9	125.0
C1^i^—Ru1—C5	90.57 (11)	C11—C10—C9	114.9 (4)
C7—Ru1—C5	61.24 (11)	C11—C10—H10	122.6
C3—Ru1—C5	61.10 (10)	C9—C10—H10	122.6
C6—Ru1—C5	36.30 (11)	C10—C11—S1	111.7 (3)
C4—Ru1—C5	36.45 (10)	C10—C11—H11	124.1
C2—Ru1—Ru1^i^	94.32 (10)	S1—C11—H11	124.1
C1—Ru1—Ru1^i^	47.87 (8)	C4—C12—H12A	109.5
C1^i^—Ru1—Ru1^i^	46.97 (8)	C4—C12—H12B	109.5
C7—Ru1—Ru1^i^	170.96 (8)	H12A—C12—H12B	109.5
C3—Ru1—Ru1^i^	140.96 (7)	C4—C12—H12C	109.5
C6—Ru1—Ru1^i^	134.95 (8)	H12A—C12—H12C	109.5
C4—Ru1—Ru1^i^	112.39 (7)	H12B—C12—H12C	109.5
C5—Ru1—Ru1^i^	109.85 (8)	C5—C13—H13A	109.5
C11—S1—C8	91.94 (18)	C5—C13—H13B	109.5
O2—C1—Ru1	139.3 (2)	H13A—C13—H13B	109.5
O2—C1—Ru1^i^	135.5 (2)	C5—C13—H13C	109.5
Ru1—C1—Ru1^i^	85.15 (10)	H13A—C13—H13C	109.5
O1—C2—Ru1	175.9 (3)	H13B—C13—H13C	109.5
C4—C3—C7	107.5 (2)	C6—C14—H14A	109.5
C4—C3—C8	126.3 (2)	C6—C14—H14B	109.5
C7—C3—C8	126.0 (3)	H14A—C14—H14B	109.5
C4—C3—Ru1	73.49 (15)	C6—C14—H14C	109.5
C7—C3—Ru1	69.90 (15)	H14A—C14—H14C	109.5
C8—C3—Ru1	126.41 (19)	H14B—C14—H14C	109.5
C3—C4—C5	108.1 (2)	C7—C15—H15A	109.5
C3—C4—C12	125.5 (3)	C7—C15—H15B	109.5
C5—C4—C12	126.3 (3)	H15A—C15—H15B	109.5
C3—C4—Ru1	70.06 (15)	C7—C15—H15C	109.5
C5—C4—Ru1	72.19 (16)	H15A—C15—H15C	109.5
C12—C4—Ru1	125.7 (2)	H15B—C15—H15C	109.5
C6—C5—C4	108.4 (3)		
			
C2—Ru1—C1—O2	−85.4 (4)	Ru1^i^—Ru1—C5—C4	−101.12 (15)
C1^i^—Ru1—C1—O2	−178.9 (5)	C2—Ru1—C5—C13	−119.8 (4)
C7—Ru1—C1—O2	12.6 (5)	C1—Ru1—C5—C13	73.4 (3)
C3—Ru1—C1—O2	25.7 (4)	C1^i^—Ru1—C5—C13	−22.3 (3)
C6—Ru1—C1—O2	71.9 (5)	C7—Ru1—C5—C13	−156.8 (3)
C4—Ru1—C1—O2	61.3 (4)	C3—Ru1—C5—C13	159.6 (3)
C5—Ru1—C1—O2	88.0 (4)	C6—Ru1—C5—C13	−119.5 (4)
Ru1^i^—Ru1—C1—O2	−178.9 (5)	C4—Ru1—C5—C13	122.6 (4)
C2—Ru1—C1—Ru1^i^	93.50 (13)	Ru1^i^—Ru1—C5—C13	21.5 (3)
C1^i^—Ru1—C1—Ru1^i^	0.0	C4—C5—C6—C7	−1.5 (3)
C7—Ru1—C1—Ru1^i^	−168.55 (12)	C13—C5—C6—C7	−175.7 (3)
C3—Ru1—C1—Ru1^i^	−155.46 (10)	Ru1—C5—C6—C7	60.44 (19)
C6—Ru1—C1—Ru1^i^	−109.3 (2)	C4—C5—C6—C14	176.4 (3)
C4—Ru1—C1—Ru1^i^	−119.83 (10)	C13—C5—C6—C14	2.2 (5)
C5—Ru1—C1—Ru1^i^	−93.11 (11)	Ru1—C5—C6—C14	−121.6 (3)
C2—Ru1—C3—C4	174.96 (17)	C4—C5—C6—Ru1	−61.97 (18)
C1—Ru1—C3—C4	78.61 (17)	C13—C5—C6—Ru1	123.9 (3)
C1^i^—Ru1—C3—C4	−40.9 (3)	C2—Ru1—C6—C7	62.3 (2)
C7—Ru1—C3—C4	−116.2 (2)	C1—Ru1—C6—C7	−92.8 (3)
C6—Ru1—C3—C4	−78.47 (17)	C1^i^—Ru1—C6—C7	157.92 (18)
C5—Ru1—C3—C4	−36.95 (16)	C3—Ru1—C6—C7	−39.01 (17)
Ru1^i^—Ru1—C3—C4	49.3 (2)	C4—Ru1—C6—C7	−80.71 (18)
C2—Ru1—C3—C7	−68.8 (2)	C5—Ru1—C6—C7	−117.6 (2)
C1—Ru1—C3—C7	−165.16 (18)	Ru1^i^—Ru1—C6—C7	−174.32 (13)
C1^i^—Ru1—C3—C7	75.4 (3)	C2—Ru1—C6—C5	179.85 (18)
C6—Ru1—C3—C7	37.75 (17)	C1—Ru1—C6—C5	24.8 (3)
C4—Ru1—C3—C7	116.2 (2)	C1^i^—Ru1—C6—C5	−84.50 (18)
C5—Ru1—C3—C7	79.27 (18)	C7—Ru1—C6—C5	117.6 (2)
Ru1^i^—Ru1—C3—C7	165.55 (13)	C3—Ru1—C6—C5	78.57 (18)
C2—Ru1—C3—C8	51.7 (3)	C4—Ru1—C6—C5	36.87 (16)
C1—Ru1—C3—C8	−44.6 (3)	Ru1^i^—Ru1—C6—C5	−56.74 (19)
C1^i^—Ru1—C3—C8	−164.1 (2)	C2—Ru1—C6—C14	−58.7 (3)
C7—Ru1—C3—C8	120.5 (3)	C1—Ru1—C6—C14	146.3 (3)
C6—Ru1—C3—C8	158.3 (3)	C1^i^—Ru1—C6—C14	36.9 (3)
C4—Ru1—C3—C8	−123.2 (3)	C7—Ru1—C6—C14	−121.0 (4)
C5—Ru1—C3—C8	−160.2 (3)	C3—Ru1—C6—C14	−160.0 (3)
Ru1^i^—Ru1—C3—C8	−73.9 (3)	C4—Ru1—C6—C14	158.3 (3)
C7—C3—C4—C5	0.5 (3)	C5—Ru1—C6—C14	121.4 (4)
C8—C3—C4—C5	−174.1 (3)	Ru1^i^—Ru1—C6—C14	64.7 (3)
Ru1—C3—C4—C5	62.55 (18)	C5—C6—C7—C3	1.8 (3)
C7—C3—C4—C12	177.7 (3)	C14—C6—C7—C3	−176.0 (3)
C8—C3—C4—C12	3.1 (4)	Ru1—C6—C7—C3	64.53 (19)
Ru1—C3—C4—C12	−120.3 (3)	C5—C6—C7—C15	174.8 (3)
C7—C3—C4—Ru1	−62.02 (18)	C14—C6—C7—C15	−3.1 (5)
C8—C3—C4—Ru1	123.4 (3)	Ru1—C6—C7—C15	−122.6 (3)
C2—Ru1—C4—C3	−8.6 (3)	C5—C6—C7—Ru1	−62.68 (19)
C1—Ru1—C4—C3	−104.80 (17)	C14—C6—C7—Ru1	119.4 (3)
C1^i^—Ru1—C4—C3	159.04 (16)	C4—C3—C7—C6	−1.5 (3)
C7—Ru1—C4—C3	38.80 (16)	C8—C3—C7—C6	173.1 (3)
C6—Ru1—C4—C3	80.91 (17)	Ru1—C3—C7—C6	−65.85 (19)
C5—Ru1—C4—C3	117.6 (2)	C4—C3—C7—C15	−174.4 (3)
Ru1^i^—Ru1—C4—C3	−148.89 (13)	C8—C3—C7—C15	0.2 (5)
C2—Ru1—C4—C5	−126.2 (2)	Ru1—C3—C7—C15	121.2 (3)
C1—Ru1—C4—C5	137.56 (18)	C4—C3—C7—Ru1	64.38 (18)
C1^i^—Ru1—C4—C5	41.4 (2)	C8—C3—C7—Ru1	−121.0 (3)
C7—Ru1—C4—C5	−78.84 (18)	C2—Ru1—C7—C6	−125.6 (2)
C3—Ru1—C4—C5	−117.6 (2)	C1—Ru1—C7—C6	136.91 (19)
C6—Ru1—C4—C5	−36.73 (17)	C1^i^—Ru1—C7—C6	−28.5 (2)
Ru1^i^—Ru1—C4—C5	93.47 (16)	C3—Ru1—C7—C6	115.6 (2)
C2—Ru1—C4—C12	111.4 (3)	C4—Ru1—C7—C6	78.44 (18)
C1—Ru1—C4—C12	15.2 (3)	C5—Ru1—C7—C6	36.77 (16)
C1^i^—Ru1—C4—C12	−81.0 (3)	C2—Ru1—C7—C3	118.77 (19)
C7—Ru1—C4—C12	158.8 (3)	C1—Ru1—C7—C3	21.3 (3)
C3—Ru1—C4—C12	120.0 (3)	C1^i^—Ru1—C7—C3	−144.14 (17)
C6—Ru1—C4—C12	−159.1 (3)	C6—Ru1—C7—C3	−115.6 (2)
C5—Ru1—C4—C12	−122.4 (3)	C4—Ru1—C7—C3	−37.20 (16)
Ru1^i^—Ru1—C4—C12	−28.9 (3)	C5—Ru1—C7—C3	−78.87 (18)
C3—C4—C5—C6	0.6 (3)	C2—Ru1—C7—C15	−2.7 (3)
C12—C4—C5—C6	−176.5 (3)	C1—Ru1—C7—C15	−100.2 (3)
Ru1—C4—C5—C6	61.80 (18)	C1^i^—Ru1—C7—C15	94.4 (3)
C3—C4—C5—C13	174.6 (3)	C3—Ru1—C7—C15	−121.5 (3)
C12—C4—C5—C13	−2.5 (4)	C6—Ru1—C7—C15	122.9 (3)
Ru1—C4—C5—C13	−124.2 (3)	C4—Ru1—C7—C15	−158.7 (3)
C3—C4—C5—Ru1	−61.19 (18)	C5—Ru1—C7—C15	159.7 (3)
C12—C4—C5—Ru1	121.7 (3)	C4—C3—C8—C9	−114.8 (4)
C2—Ru1—C5—C6	−0.3 (3)	C7—C3—C8—C9	71.6 (5)
C1—Ru1—C5—C6	−167.05 (16)	Ru1—C3—C8—C9	−18.8 (5)
C1^i^—Ru1—C5—C6	97.23 (18)	C4—C3—C8—S1	69.0 (3)
C7—Ru1—C5—C6	−37.27 (17)	C7—C3—C8—S1	−104.6 (3)
C3—Ru1—C5—C6	−80.92 (18)	Ru1—C3—C8—S1	164.94 (16)
C4—Ru1—C5—C6	−117.9 (2)	C11—S1—C8—C9	1.7 (3)
Ru1^i^—Ru1—C5—C6	141.01 (15)	C11—S1—C8—C3	178.6 (3)
C2—Ru1—C5—C4	117.6 (3)	C3—C8—C9—C10	−178.1 (3)
C1—Ru1—C5—C4	−49.17 (19)	S1—C8—C9—C10	−1.6 (4)
C1^i^—Ru1—C5—C4	−144.90 (17)	C8—C9—C10—C11	0.6 (5)
C7—Ru1—C5—C4	80.60 (18)	C9—C10—C11—S1	0.7 (5)
C3—Ru1—C5—C4	36.96 (16)	C8—S1—C11—C10	−1.4 (4)
C6—Ru1—C5—C4	117.9 (2)		

Symmetry codes: (i) −*x*, −*y*+1, −*z*+1.

### Hydrogen-bond geometry (Å, °)

**Table:**

*D*—H···*A*	*D*—H	H···*A*	*D*···*A*	*D*—H···*A*
C10—H10···O2^ii^	0.93	2.60	3.335 (5)	136
C14—H14B···O2^i^	0.96	2.58	3.319 (4)	134

Symmetry codes: (ii) −*x*+1, −*y*, −*z*+1; (i) −*x*, −*y*+1, −*z*+1.
